# Enhanced catecholamine transporter binding in the locus coeruleus of patients with early Parkinson disease

**DOI:** 10.1186/1471-2377-11-88

**Published:** 2011-07-21

**Authors:** Ioannis U Isaias, Giorgio Marotta, Gianni Pezzoli, Osama Sabri, Johannes Schwarz, Paolo Crenna, Joseph Classen, Paolo Cavallari

**Affiliations:** 1 Università degli Studi di Milano, Dipartimento di Fisiologia Umana, Milano, Italy; 2 Parkinson Institute, Istituti Clinici di Perfezionamento, Milano, Italy; 3 Department of Neurology, University of Leipzig, Leipzig, Germany; 4 Department of Nuclear Medicine, Fondazione IRCCS Ca' Granda - Ospedale Maggiore Policlinico, Milano, Italy; 5 Department of Nuclear Medicine, University of Leipzig, Leipzig, Germany

## Abstract

**Background:**

Studies in animals suggest that the noradrenergic system arising from the locus coeruleus (LC) and dopaminergic pathways mutually influence each other. Little is known however, about the functional state of the LC in patients with Parkinson disease (PD).

**Methods:**

We retrospectively reviewed clinical and imaging data of 94 subjects with PD at an early clinical stage (Hoehn and Yahr stage 1-2) who underwent single photon computed tomography imaging with FP-CIT ([^123^I] N-ω-fluoropropyl-2β-carbomethoxy-3β-(4-iodophenyl) tropane). FP-CIT binding values from the patients were compared with 15 healthy subjects: using both a voxel-based whole brain analysis and a volume of interest analysis of *a priori *defined brain regions.

**Results:**

Average FP-CIT binding in the putamen and caudate nucleus was significantly reduced in PD subjects (43% and 57% on average, respectively; p < 0.001). In contrast, subjects with PD showed an increased binding in the LC (166% on average; p < 0.001) in both analyses. LC-binding correlated negatively with striatal FP-CIT binding values (caudate: contralateral, ρ = -0.28, p < 0.01 and ipsilateral ρ = -0.26, p < 0.01; putamen: contralateral, ρ = -0.29, p < 0.01 and ipsilateral ρ = -0.29, p < 0.01).

**Conclusions:**

These findings are consistent with an up-regulation of noradrenaline reuptake in the LC area of patients with early stage PD, compatible with enhanced noradrenaline release, and a compensating activity for degeneration of dopaminergic nigrostriatal projections.

## Background

The pontine nucleus locus coeruleus (LC) is the major site of noradrenaline (NA) neurons in the central nervous system, hosting almost half of the NA-producing neurons in the brain [1].

The LC may play an important role in the pathophysiology of Parkinson disease (PD) for several reasons: (i) as a site of neuronal degeneration as part of PD pathology; [2] (ii) as the anatomical origin of projections modulating dopaminergic action of the substantia nigra; [3] (iii) as a structure under putative dopaminergic inhibitory control from the ventral tegmental area (VTA) which is known to degenerate in PD [4,5]. Based on physiological functions ascribed to the noradrenergic system, impaired functioning of LC in PD has been associated primarily to affective disorders, [6] cognitive disturbances, [7] sleep disorders, [8] sensory impairment [2] and autonomic dysfunction [9]. Through its interactions with the dopaminergic system however, the LC may also have a less direct role in the pathogenesis of PD via (i) an interplay of catecholamine systems with one amine cross-talking with receptors belonging to the other system [10,11] or (ii) extra-synaptic neuro-modulatory, metabotroic and trophic activities of noradrenaline itself [12].

Information on the LC in PD is mainly based on post-mortem examination of histopathological specimens, while information on its *in vivo *function is largely absent. Ideally, the LC-NA system and noradrenaline molecular transporters (NET) should be investigated *in vivo *by dedicated, highly specific radiotracers displaying low background non-NET binding, high sensitivity to variations in NET density and fast kinetics. As such a radiotracer is not available for large clinical studies, [13] we employed single photon computed tomography (SPECT) with FP-CIT ([^123^I] N-ω-fluoropropyl-2β-carbomethoxy-3β-(4-iodophenyl) tropane) in a large, homogeneous cohort of early stage PD patients. Although FP-CIT is mainly used for assessing striatal dopamine reuptake transporters, it has shown sensitivity, albeit lower, to NET [14]. Therefore, when applied to an anatomical region with known low dopamine reuptake transporter capacity such as the LC, it allows investigation of the NA-dependent synaptic activity.

## Methods

### Subjects

We retrospectively reviewed clinical and imaging data of 94 subjects with idiopathic PD in whom FP-CIT SPECT was performed at the "Ospedale Maggiore Policlinico" in Milano within five years of the onset of motor symptoms. Fifteen healthy subjects (healthy controls, HC) were prospectively enrolled for comparisons of FP-CIT binding. At the time of SPECT, HC did not suffer from any disease and were not taking any medications. Clinical inclusion criteria for subjects with PD were: (a) diagnosis according to the UK Parkinson Disease Brain Bank criteria; (b) absence of any signs indicative for atypical parkinsonism (e.g. gaze abnormalities, autonomic dysfunction, significant psychiatric disturbances, etc.) over a follow-up period of at least three years after symptoms onset; (c) Hoehn and Yahr (H&Y) stage 1 or 2 in drugs-off state (i.e. after overnight withdrawal of specific drugs for PD; no patients were taking long-acting dopaminergic drugs) at the time of SPECT; (d) positive clinical improvement at Unified Parkinson Disease Rating Scale (UPDRS) after L-Dopa intake (i.e. > 30% from drug-off state) at some point during the three years of follow up; (e) a normal Magnetic Resonance Imaging (MRI) (no sign of white matter lesion or atrophy). Finally, given a putative role of LC and noradrenaline in cognition and mood (including depression) we excluded from this study patients with a positive score at UPDRS part I.

A quantitative profile of each patient' motor impairment was obtained from clinical assessment performed before SPECT by means of the UPDRS motor part (part III). L-Dopa daily dose and L-Dopa Equivalent Daily Doses (LEDDs) were also recorded, with the latter expressed as follows: 100 mg levodopa = 1.5 mg pramipexole = 6 mg ropinirole. None of the subjects (both PD and HC) were taking or stated to have ever been treated with antipsychotics or drugs known to affect the noradrenergic system (e.g., noradrenaline reuptake inhibitors). Drug naïve patients at the time of SPECT were not included in the study. The Ethics Committee of the Department of Human Physiology approved the study and all subjects gave informed consent.

### SPECT data acquisition and processing

Intravenous administration of 110-140 MBq of FP-CIT (DaTSCAN, GE-Healthcare, UK) was performed 30-40 minutes after thyroid blockade (10-15 mg of Lugol oral solution) in the control subjects and in patients after overnight withdrawal of dopaminergic therapy [15]. Brain SPECT was performed 3 hours later by means of a dedicated triple detector gamma-camera (Prism 3000, Philips, Eindhoven, the Netherlands) equipped with low-energy ultra-high resolution fan beam collimators (4 subsets of acquisitions, matrix size 128x128, radius of rotation 12.9-13.9 cm, continuous rotation, angular sampling: 3 degree, duration: 28 minutes). Brain sections were reconstructed with an iterative algorithm (OSEM, 4 iterations and 15 subsets) and then processed by 3D filtering (Butterworth, 5^th ^order, cut-off 0.31 pixel^-1^) and attenuation correction (Chang method, factor 0.12).

### Imaging data analysis

Two different and complementary image analyses were performed: a voxel-based whole brain analysis using Statistical Parametric Mapping SPM2 (Wellcome Department of Imaging Neuroscience, London, UK) implemented in MATLAB R2007a (The Mathworks Inc, USA), and a volume of interest (VOI) analysis of *a priori *defined brain regions.

#### SPM analysis

A group-specific FP-CIT template was created by (i) spatially normalizing the FP-CIT images of 15 healthy subjects onto a FP-CIT MNI-based template, [16] (ii) subsequent averaging of the normalized images and their symmetric (mirror) images resulting in a mean image, and finally (iii) a smoothing of the mean image using a 3-dimensional Gaussian kernel with 8-mm full width at half maximum (FWHM). To increase the signal-to-noise ratio and account for subtle variations in anatomic structures, the individual subject's FP-CIT images were spatially normalized to this group-specific template and smoothed with a FWHM 10-mm Gaussian kernel. A reference region in the occipital cortex was defined as the union of the superior, middle and inferior occipital gyri along with the calcarine gyri VOIs defined by automated anatomical labelling (AAL), using the Wake Forest University (WFU) PickAtlas 2.4 software. Binding values for each subject's FP-CIT image were then computed in a voxel-by-voxel manner (voxel - occipital reference)/(occipital reference). Using the General Linear Model in voxel-based statistical analysis of SPM2, a two-sample t-test contrast was used to elucidate group difference between PD and HC. No global normalization, or grand mean scaling, were applied, and the masking threshold was set to zero. Clusters of at least 35 voxels with the height threshold set at p < 0.001, were considered significant.

#### VOI analysis

The LC FP-CIT binding values were for two VOIs (for left and right part of LC) created, using WFU Pick Atlas Tool, through the union of six distinct, contiguous Boxes (of 3 mm on the *z *axis for each side), centered in the mean values on the *x *and *y *axis and dimensioned according to the standard deviation as proposed in Table 1 of Keren and coll., 2009 [17]. FP-CIT binding values for the caudate nucleus (CN) and putamen (PT) were calculated on the basis of VOIs defined with the Basal Ganglia Matching Tool [18]. Student's *t-*test was then applied. We defined as *contralateral*, those brain regions opposite to that of PD most severe sign presentation. For HC, we referred to the right side as *ipsilateral *[15].

**Table 1:** Demographics and clinical data

	PD	HC
Subjects N. (male/female)	94 (67/27)	15 (4/11)*

Age at SPECT	60 (38 - 75)	63 (51 - 74)

Age at motor symptoms onset	57 (37 - 72)	

Disease duration	3 (1 - 5)	

UPDRS motor score (part-III) [range 0 - 108]	19 (8 - 56)	

Hoehn and Yahr stage [range 1 - 5]	2 (1 - 2)	

L-Dopa in mg/day	400 (0 - 850)	

LEDDs in mg/day	250 (70 - 1200)	

Data are reported as median and range (brackets). Age at SPECT, age at motor symptoms onset and disease duration are in years. All patients were evaluated with the Unified Parkinson Disease Rating Scale motor part (UPDRS part III) in drugs-off state (i.e. after overnight withdrawal of specific drugs for PD, no patients was taking long-acting dopaminergic drugs). LEDDs were calculated as follow: 100 mg levodopa = 1.5 mg pramipexole = 6 mg ropinirole. * p = 0.0005.

### General statistical analysis

Unless otherwise stated, data are reported as median and range. Normality of data distribution was tested by means of Shapiro-Wilks test. Chi-Square was used to test gender distribution among groups. Demographic data were compared by means of Wilcoxon two-group test. The Spearman correlation coefficient was calculated to investigate statistical dependence among average binding values, demographic and clinical variables.

Statistical analyses were performed with the JMP statistical package, version 8.0 (SAS Institute, Inc., Cary, NC, USA).

## Results

Table 1 shows the demographic and clinical characteristics of the study cohorts.

**Figure 1:**
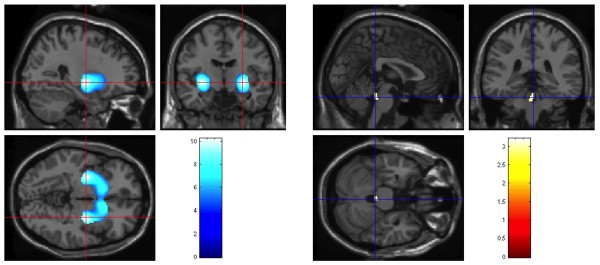
**Overlay on a MRI showing the loss of FP-CIT binding bilaterally in the striatum (cluster of 6819 voxels, peaks at coordinates: 28 -8 -4 and at -31 -8 -4) (left in the figure) and increased FP-CIT binding in the locus coeruleus area (cluster of 37 voxels, peak at coordinates: 2 -36 -26) (right in the figure) of the whole group of PD patients compared to controls**.

SPM analysis detected one large cluster of 6819 voxels (peaks at coordinates: 28 -8 -4 and at -31 -8 -4) of significantly reduced FP-CIT binding involving the PT and the CN bilaterally (Figure 1, left). A cluster of 37 voxels (peak at coordinates: 2 -36 -26) with higher FP-CIT binding values was found in the LC of PD subjects (Figure 1, right).

Volumes of interest analysis revealed reduced average binding values in the striatum and increased average binding value in LC area, bilaterally (Table 2).

**Table 2:** Binding values obtained with the analysis of volumes of interest

Region of interest	PD	HC	p values
CN contralateral	3.18 (1.2 - 3.84)	5.27 (3.51 - 6.15)	< 0.0001

CN ipsilateral	3.29 (0.98 - 6.26)	5.27 (3.51 - 6.6)	< 0.0001

PT contralateral	1.86 (0.65 - 4.72)	4.83 (3.07 - 6.04)	< 0.0001

PT ipsilateral	1.97 (0.76 - 4.65)	4.83 (3.62 - 6.37)	< 0.0001

LC contralateral	313.5 (81 - 663)	131.43 (59 - 371)	0.001

LC ipsilateral	321.17 (87 - 632)	123.6 (38 - 354)	0.0004

Data are reported as median and range (brackets). Average FP-CIT binding in the caudate nucleus (CN) and putamen (PT) was significantly reduced in subjects with PD subjects compared to HC. On the contrary, PD patients showed a significantly increased binding in the LC area (both right and left regions). We defined *contralateral *brain regions opposite to that of PD signs presentation. For HC, we referred to the right side as *ipsilateral *[15].

**Figure 2:**
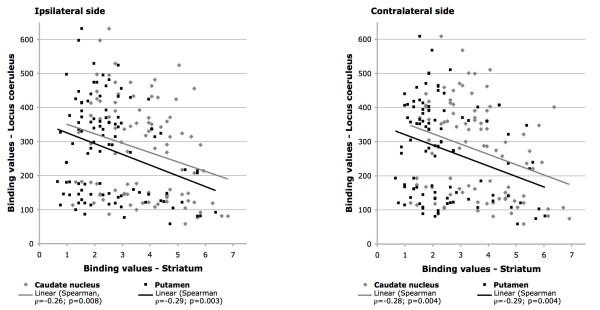
**Scatter plots and linear correlations of ipsilateral (left in the figure) and contralateral (right in the figure) FP-CIT binding values of the locus coeruleus and striatum (caudate nucleus and putamen)**. A statistically significant negative correlation was found between FP-CIT binding values in the locus coeruleus area and the corresponding striatum (both caudate nucleus and putamen).

FP-CIT binding in the striatum showed a weak, but significant, negative correlation with binding values of the corresponding LC (caudate: contralateral, ρ = -0.28, p = 0.004 and ipsilateral ρ = -0.26, p = 0.008; putamen: contralateral, ρ = -0.29, p = 0.004 and ipsilateral ρ = -0.29, p = 0.003) (Figure 2). LC binding did not show other significant correlations. Finally, results for the FP-CIT binding value in the LC area proved to be statistically independent when weighted for demographic (age at SPECT, age at onset, gender) and clinical characteristics (disease duration, disease severity and L-Dopa daily dose and LEDDs).

## Discussion

### Increased FP-CIT binding in the LC area

The present study provides *in vivo *evidence of higher baseline catecholamine transporter binding in the LC region in a large and homogeneous cohort of subjects with early PD. Our findings are consistent with an up-regulation of noradrenaline reuptake in the LC area, which is well compatible with enhanced noradrenaline release [19,20].

Our results are derived from the analysis of the binding of FP-CIT, a ^123^I-labeled cocaine derivative with high affinity for dopamine (DAT; K_D _= 2nM) and a lesser affinity towards noradrenaline transporter (NET; K_D _= 140 nM) [14]. Despite the higher affinity of FP-CIT for DATs, it is unlikely that the higher binding observed in the LC area is due to an enhanced dopaminergic, rather than noradrenergic, transporter for two main reasons: (i) in LC, DAT represent a minor and inconsistent component of the midbrain-derived dopaminergic terminals which degenerates in PD, along with other dopaminergic projections, [4] and (ii) in the LC a major NET component is synthesized in the cell body of pigmented neurons and exposed on their membrane to be transferred toward axonal terminals, [20] with a less consistent NET component localized on terminal projections arising from more caudal noradrenergic cell groups [21].

In PD, reduced dopamine release from nigro-striatal projections results in loss and adaptive down-regulation of DAT binding sites in the striatal region [22]. In line with this notion, and in agreement with studies in *de novo *and early PD, where 40 to 60% of nigral dopaminergic neurons are lost, [15,23] we found a significantly reduced FP-CIT binding in the caudate and putamen of PD patients. In contrast to the striatal compartment, analysis of FP-CIT labeling in the upper brainstem revealed significantly increased binding in a pontine area adjacent to the floor of the fourth ventricle and extending into the midbrain to the level of the inferior colliculi. This area corresponds topographically to the LC coordinates identified by other studies including those employing neuromelanin-sensitive MRI methods [6,17,24,25]. In addition, the LC is the sole structure in the posterior rostral pons housing monoamine transporters [1], thus further supporting our claim of anatomical targeting of the LC.

Only two prior studies with PET have specifically investigated the LC in PD patients. A first, [24] reported a reduced ^18^F-dopa intake in patients with advanced PD when compared to patients at an early stage of the disease. Because ^18^F-dopa intake is more specifically related to dopaminergic neurotransmission, this study does not provide information on noradrenergic functioning of LC. In a second study, [6] PD subjects with depression showed a reduced binding of [^11^C]RTI-32, a marker of both DAT and NET, when compared to non-depressed patients. Interestingly, the noradrenergic activity of early non-depressed PD patients was within normal range in most patients and enhanced in few of them. In line with these findings, and having enrolled a larger and more selected cohort of subjects, we were able to reveal a significantly higher LC activity at an early stage of PD for the first time.

An acute effect of drugs on FP-CIT binding values appears unlikely since SPECT was performed after overnight withdrawal of anti-parkinsonian drugs. In addition, in animal studies, systemic administration of D_2_/D_3 _receptor agonists, such as pramipexole or apomorphine, showed little or no effect on the firing rate of LC-NA neurons [26]. Finally, a persistent treatment with dopaminergic drugs will eventually down-regulate, rather than up-regulate, the surface expression of DAT and NET through internalization of the transporters [27,28]. Accordingly, the average FP-CIT binding values in the LC remained enhanced when data were L-Dopa weighted for equivalent daily dose and L-Dopa daily dose.

### *In vivo *versus anatomopathological studies

Enhanced noradrenergic binding, and possibly activity, in PD might be considered at odds with neuropathological findings, where frank neuronal degeneration has been recognized within LC, based on detection of specific cellular markers. Indeed, morphologic hallmarks of sporadic PD (Lewy bodies and dystrophic neurites containing pathologic α-synuclein) may appear initially in the lower brainstem [2].

However, Lewy pathology can correlate poorly with neuronal loss in specific areas, thus its validity in predicting neuronal disintegration is questionable [29]. In fact, noradrenergic neurons in the LC are relatively preserved in early PD and do not exhibit the same intracellular changes as in the substantia nigra [30].

Accordingly, neuromelanin-sensitive imaging methods *in vivo*, [25] as well as anatomopathological studies suggested that the loss of NA neurons in PD may be confined to the larger, pigmented cells localized in the caudal part of the nucleus, whereas small unpigmented cells are increased in number, as if derived from shrinkage of larger neurons [31].

However, available information on the LC, so far derived from anatomopathological studies in subjects with PD, is poorly comparable with our findings. In particular, the limited number of PD subjects investigated and the lack of clinical information (e.g. disease duration and the presence of depression or cognitive impairment) of patients in anatomopathological studies prevent a direct comparison between these studies and our results [31,32].

### Implications of enhanced LC-NA functioning in PD at an early stage

Based on anatomical and histochemical data, along with neuropharmacological evidence, higher activity of the LC in PD may suggest: (i) in the striatum, noradrenaline plays a compensatory role cross targeting dopaminergic receptors (synaptic action); while (ii) in the substantia nigra, noradrenaline has a neuroprotective bolstering dopaminergic cells (extra-synaptic paracrine action).

As for the compensatory role, there is no absolute specificity for catecholaminergic substrate-receptor interactions, implying that one catecholamine can cross-talk with the pharmacologically defined receptors or transporters belonging to other catecholamines. Indeed, noradrenaline binds to pharmacologically defined dopaminergic receptors [11,33,34]. Therefore, enhanced noradrenaline release may be able to partially compensate a dopaminergic innervation loss due to degeneration of the substantia nigra.

With reference to a putative neuroprotective activity, noradrenaline suppresses pro-inflammatory and elevates anti-inflammatory molecules [35] and has the ability to scavenge superoxide and reactive oxygen species, which are thought to contribute to cellular damage and dopaminergic cell death [36]. Furthermore, the tottering mouse, which has noradrenergic hyperinnervation and increased levels of noradrenaline throughout the forebrain, appears to be protected from MPTP toxicity [37] while MPTP-induced damage to nigrostriatal dopaminergic neurons was potentiated by pretreatment with DSP-4, a selective LC neurotoxin [38]. Therefore, we speculate that enhanced LC-NA may be regarded as an endogenous paracrine agent promoting dopaminergic neuron survival [39,40]. This hypothesis would predict that degeneration of LC noradrenergic neurons in later stages of the disease might accelerate degeneration of substantia nigra dopaminergic neurons. The negative correlation between FP-CIT binding in the striatum and LC area is consistent with the above considerations of LC-NA compensatory and protective activity.

## Conclusions

The present study suggests higher baseline catecholamine transporter binding in the LC area of patients with early stage PD. We propose that enhanced noradrenergic activity may be one factor modulating the severity of motor symptoms and may even influence progression of dopaminergic neurodegeneration.

## Competing interests

The authors declare that they have no competing interests.

## Authors' contributions

IUI and GM participated in the conception of the study, gathered and analyzed the data. JC, PC, GP, OS, JS and PC contributed to data analysis and participated in the redaction of the paper. All authors read and approved the final manuscript.

## Pre-publication history

The pre-publication history for this paper can be accessed here:

http://www.biomedcentral.com/1471-2377/11/88/prepub
